# Editorial: Identifying novel drug delivery systems and treatments for hearing loss and related ear disorders, volume II

**DOI:** 10.3389/fphar.2024.1464254

**Published:** 2024-07-29

**Authors:** Sho Kanzaki, Taro Yamaguchi, Myung-Whan Suh

**Affiliations:** ^1^ National Institute of Sensory Organ, National Hospital Organization of Tokyo Medical Center, Meguro City, Japan; ^2^ Faculty of Pharmaceutical Sciences, Setsunan University, Hirakata, Japan; ^3^ Department of Otorhinolaryngology-Head and Neck Surgery, Seoul National University Hospital, Seoul, Republic of Korea; ^4^ Sensory Organ Research Institute, Seoul National University Medical Research Center, Seoul, Republic of Korea

**Keywords:** inner ear, drug delivery, therapy, hearing loss, presbycusis

Development of drug delivery system (DDS) for the middle ear is a useful way to treat otitis media, which is still a very common disease.

Furthermore, the development of DDS for the inner ear is a very important, as is drug development for the inner ear. No matter how good a drug is, if it does not reach the inner ear, it has no clinical significance. On the other hand, even drugs with weak efficacy may be clinically effective if they reach the inner ear in large doses.

Most therapeutic drugs are administered orally or by injection, but the inner ear contains blood labyrinth barriers (BLBs), which are often difficult to reach by administration through the blood. Local administration or intratympanic injection (ITI) of drug to the inner ear is therefore considered, but the inner ear is anatomically surrounded by bone and has a barrier in the inner ear membrane. Additionally, because administering drugs through a hole in the inner ear membrane can cause mechanical damage, we also consider whether the drug can penetrate the barrier. Furthermore, we administer the drug slowly over a long period because the volume of inner ear is very small.

In this Research Topic, we have six following articles addressing the above issues.

**FIGURE 1:**
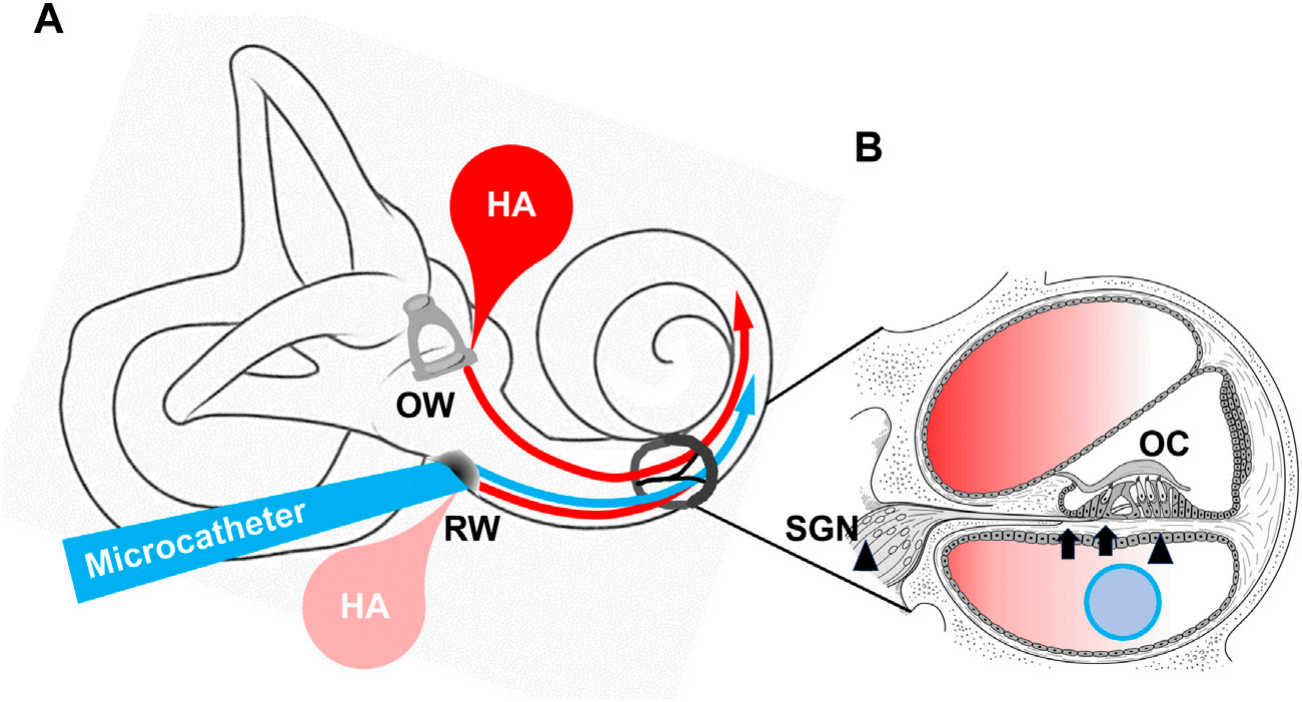
A. Drug delivery into cochlea Arrows indicate microcatheter and hyaluronic acid target inner ear. RW; round window OW; oval window B. Section of cochlea ELZC treatment target (arrows), and resveratrol treatment target (arrowheads) in presbycusis, respectively. OC; organ of Corti, SGN; spiral ganglion neuron.

1) Two articles discussing methods of inner ear administration, 2) two articles on intra-tympanic steroid administration, which is currently used in clinical practice, and 3) two articles attempting to improve age-related hearing loss (ARHL) or presbycusis (Figure 1).

The novel developments in drug delivery for middle and inner ear administration including novel controlled release therapeutics such as hydrogel and nanotechnology and finally, novel device delivery approaches such as microfluidic systems and cochlear prosthesis-mediated delivery (Delaney et al.).

Because the size of the inner ear differs between humans and other animals, it is questionable whether the results of nonclinical studies can be applied to humans, however, pharmacokinetics in catheterized piglets were evaluated using fluorescent dyes. Fluorescent dyes concentrations were significantly lower 6 h and 24 h compared to 2 h after administration (Yildiz et al.). We may need constant administration or vehicles that are designed to resist drainage through the Eutachian tube and enable sustained-release.

Intratympanic injection (ITI) of steroids is also used clinically, mainly for sudden senosorineural hearing loss, to reduce the side effects of systemic steroid administration, but ITI with multiple tympanostomy carries the risk of perforation of the tympanic membrane. Two strategies were therefore proposed on this Research Topic.

High-molecular-weight hyaluronic acid (HHA) is prolongs residence time, enhances drug concentration in target tissues, and ensures safety, highlighting the potential advantages of HHA + Dexamethasone over existing standard therapies (Hwang et al.).

ITI of steroid with embedded dual viscosity mixture vehicle (DVV) increases efficacy, and this activity is reflected in reduced ROS activity, inflammatory response, and actin filament-mediated wound healing processes (Jung et al.).

Finally, this Research Topic also included prevention of ARHL which is the most common form of inner ear diseases. The protective effects of ErLong ZuoC were predicted to be associated with cellular senescence, inflammatory response, and synaptic connections *in vitro* (Yang et al.). The other is an oral treatment in mice, low-dose resveratrol inhibited RIPK3-mediated necroptosis in aging cochlea and delayed the onset of ARHL, which was a promising therapeutic strategy for ARHL (Liu et al.).

In the future, many of the newer therapies for inner ear diseases will be topically administered. New drugs that can prevent ARHL without long-term medication are also expected.

